# Defining Extreme Sport: Conceptions and Misconceptions

**DOI:** 10.3389/fpsyg.2018.01974

**Published:** 2018-10-18

**Authors:** Rhonda Cohen, Bahman Baluch, Linda J. Duffy

**Affiliations:** ^1^The London Sports Institute, Faculty of Science and Technology, Middlesex University London, London, United Kingdom; ^2^Department of Psychology, Faculty of Science and Technology, Middlesex University London, London, United Kingdom

**Keywords:** extreme sport, sport, high risk sport, defining sport, thrill seeking, action sport, adventure sport, BASE jumping

## Abstract

One feature of how sport is defined is the distinction between extreme and non-extreme sport. BASE jumping is an example of an “extreme sport” because it involves a high degree of risk, whilst swimming is classified as “non-extreme” because the risks involved are minimal. This broad definition falls short of identifying the extent of risk and ignores the psychological, social-demographic and life style variables associated with engagement in each sport.

## Introduction

Indeed, the lack of consistency within the term “extreme sport" means that those wishing to study this field are forced to create their own criteria as a starting point, often in a less than scientific manner. This literary review of contemporary and historical research articles raises the key question of whether the definition of extreme sport is one of risk-taking with a high chance of injury or death or whether there are additional aspects to consider such as lifestyle or a relationship to the natural environment. This review does not examine any hypotheses and is a narrative based on key papers. Due to the lack of literature on this subject area it was not thought pertinent to conduct a systematic review.

The aim of this article is twofold: firstly, to demonstrate whether the term “extreme sport” in scientific terms, has developed into a misnomer, misleading in the context of the sports it tends to encompass, secondly, to propose a revised, more accurate definition of extreme sport, reflective of the activities it encompasses in the context of other non-mainstream sports. Based on this review it is argued that a new definition of an extreme sport is one of “a (predominantly) competitive (comparison or self-evaluative) activity within which the participant is subjected to natural or unusual physical demands. Moreover, an unsuccessful outcome is “likely to result in the injury or fatality of the participant, in contrast to non-extreme sport” (Cohen, 2016, p. 138).

## “Extreme Sport” – Challenging the Definition

The question of what is an extreme sport and whether the term “extreme sport” should be used to label particular sports can be viewed from a variety of angles. “Extreme sport” appears to be used interchangeably with “high risk sport” in much of the research literature. Both “high risk” and “extreme sport” are defined as any “sport where one has to accept a possibility of severe injury or death as an inherent part of the activity” (Breivik et al., 1994). In the same manner, classification of extreme or high risk could partly be due to peak static and dynamic components achieved during competition (Mitchell et al., 2005), which may result in bodily changes such as high blood pressure (e.g., Squash vs. Archery). A further classification would consider physical risk (e.g., BASE Jumping vs. Darts) as a defining feature of any “extreme or high risk sport” (Palmer, 2002). However, the implication that those who engage in extreme sport are exclusively high-risk taking participants is an over simplification which requires careful consideration. Part of the difficulty in being able to define extreme sport is, according to Kay and Laberge (2002). There are so many contradictory factors aside from risk. It is suggested here that there are spatial, emotional, individualistic and transgressive dimensions to consider in these sports. Terms such as “alternative,” “action,” “adventure,” and “lifestyle” are also used to describe extreme sport, however, none of these terms categorically encompass what extreme sport actually entails.

## What is Extreme?

According to Merriam-Webster’s online dictionary (retrieved September 2018) the word extreme means: (1) Exceeding the ordinary, usual or expected. (2) Existing in a very high degree. (3) Going to a great or exaggerated lengths. Therefore, extreme as used in “extreme sport” suggests a deviation beyond what is generally viewed as “normal” or “traditional” activity and assumes participants pursue activities beyond these limits. The online Oxford University Dictionary (2018) defines “extreme sport” as “Denoting or relating to a sport performed in a hazardous environment and involving great risk.” So, the concept of “going beyond normal limits” and “risk” seem integral to what constitutes extreme sport. Booker (1998) stated that “extreme sports” were beyond the boundary of moderation; surpassing what is accounted for as reasonable – i.e., radical, and sports that are located at the outermost. Breivik et al. (1994) defined extreme sport’ as a high-risk sport where the possibility of severe injury or death is a possibility as well as integral to the sport or activity. So, the components of these definitions include: going beyond the norm of what is considered reasonable and may result in severe injury or death, i.e., high physical and/or psychological risk.

## What is Sport?

Historically the definitions of sport have evolved particularly as new activities such as “BASE jumping” and “extreme mountain ironing” have emerged to challenges the perception of what sport actually is. Eysenck et al. (1982), in their seminal review paper began by highlighting the problems inherent in the definition of sport. They used the Collins dictionary in their paper to define sport as amusement, diversion, fun, pastime, game… individual or group activity pursued for exercise or pleasure often involving the testing of physical capabilities… (Eysenck et al., 1982). Arguably, this type of definition is overly inclusive, incorporating activities of amusement and pleasure whereby virtually anything that is non-work could be considered sport.

A more recent definition of sport is “all forms of physical activity which, through casual or organised participation, aimed at expressing or improving physical fitness and mental well-being, forming social relationships or obtaining results in competition at all levels” (Council of Europe [CEE], 2001, The European Sports Charter, revised, p. 3 – CEE). This broad definition of sport can encompass “traditional” sports such as Archery, Football, and Cricket, as well as those hitherto regarded as extreme sports such as Drag racing, BASE Jumping and Snowboarding.

Historically the CEES’s definition is not entirely new as sport has traditionally been accepted to represent a competitive task or activity engaged in by an individual or a group, which requires physical exertion and is governed by rules. Mason (1989) saw sport as “a more or less physically strenuous, competitive, recreational activity…usually…in the open air (which) might involve team against team, athlete against athlete or athlete against nature, or the clock.” Sport is generally viewed to be performed by individuals or in a group, as an organised, evaluative activity where the outcome of performance is judged by winning or losing. However, the inclusion of the word “or” in the CEES definition changes the nature of what is considered to be sport. It implies that results in competition do not need to be present and can be self evaluative or competitive. The modification of this definition allows activities such as recreational swimming or bungee jumping to now be classified as sports.

## Is “Extreme Sport” the Same as “High Risk Sport?”

If “extreme sport” is the same as a “high-risk” sport then those individuals that engage in these sports should be at greater risk of injury or even death than those engaging in traditional sports (Yates, 2015). When investigating the available statistics relating to extreme sport, one comes across a minefield of contradictions as the classification of injuries and/or fatalities are reported in a myriad of different ways.

A further challenge is then to set parameters using statistics of extreme sport according to risk, injury or mortality. This would require traditional sports such as cheerleading and horse riding, due to their high annual incidence of catastrophic injuries, to be classified as high-risk sports (Turner and McCory, 2006). In the United Kingdom the Rugby Football Union defined injury as something that “…prevents a player from taking a full part in all training activities typically planned for that day…” (p. 7 in the England Professional Rugby Injury Surveillance Project Season, England Professional Rugby Injury Surveillance Project Season). Mean injuries per match for 2013 were identified as 62 and mean injuries per club (including training) were 35 (p. 6 England Professional Rugby Injury Surveillance Project Season, England Professional Rugby Injury Surveillance Project Season). Annual Rugby Union incidents around the world account for 4.6 catastrophic injuries per 100,000 each year, e.g., the risk of sustaining a catastrophic injury in Rugby Union in England (0.8/100,000 per year) are relatively lower than in New Zealand (4.2/100,000 per year), Australia (4.4/100,000 per year), and Fiji (13/100,000 per year). The risk of sustaining a catastrophic injury in other contact sports are; Ice Hockey (4/100,000 per year), Rugby League (2/100,000 per year), and American Football (2/100,000 per year) (Gabbe et al., 2005; Fuller, 2008).

Besides mortality as a relevant and possible outcome, the link between the “extreme” nature of sport and brain damage arguably should be considered. Recently, the association between contact sports such as American Football and Rugby, combat sports such as Boxing and the team sport of Soccer (which includes heading balls), has resulting in a raised awareness of the relationship between sport and brain injuries and/or cognitive disturbance such as that found in Dementia. Negative effects on neuro-functioning in terms of cerebral blood flow, resulting in poor cognitive performance, can be prevalent in several sports, e.g., there have been recommendations from scuba diving research which suggested that scuba diving should be classified as a high-risk sport for the purpose of subjecting it to tighter controls and increased medical advice (Slosman et al., 2004). Alternative research suggests that classifying a sport as “extreme” should be based solely by mortality rate (Schulz et al., 2002). Mortality figures (see **Table 1**) show that whilst BASE Jumping has an extremely high mortality rate so does boxing and, somewhat surprisingly, canoeing. One may argue that employing such methods to classify sports is anything but straightforward, moreover many of the sports currently viewed as “traditional” may need further consideration as to how they could fit into a proposed working definition of extreme sport.

**Table 1:** Categorising extreme sport.

Descriptive components	1. Competitive or self evaluative	2. Natural environment (speed, height, depth, natural forces)	3. High risk evidenced by mortality statistics
**Extreme sport**			
Archery	Yes	No	No
Base jumping	Yes	Yes	1:60
Basketball	Yes	Not really	No
Boxing	Yes	Some speed	1:2,200
Canoeing (white water canoeing)	Yes	Yes	1:10,000
Cycling	Yes	Not really	1:140,845
Drag racing	Yes	Yes	484 deaths 2010–2016^∗^
Grand prix racing	Yes	Yes	1:100
Hand gliding	Yes	Yes	1:560
Motor cycle racing	Yes	Yes	1:1000
Mountain climbing	Yes	Yes	1:750
Scuba diving	Not necessarily	Usually	1:34,400
Snow boarding	Yes	Yes	1:2.2 million
Soccer	Yes	Not really	No
Swimming	Yes	Somewhat – e.g., open water endurance	1:1 million

Besides physical risk May and Slanger (2000) suggest there is potentially psychological risk when engaging in high risk sport. Their findings suggest such activities can be psychologically damaging leading to elevated stress levels, extreme competitiveness and excessive perfectionism. In view of this it could be pertinent to consider the tenets of high-risk sport as both physical and psychological. In a somewhat provocative statement, Slanger and Rudestam (1997) cited extreme sport as an expression of a death wish, whereby in a slightly different manner, Brymer and Oades (2009) considered extreme sport not to be about the expression of risk but rather about the experience of approaching danger. It is also evident that many researchers conducting studies into sensation seeking have used the term “high-risk” interchangeably with “extreme sport” (e.g., Cronin, 1991; Gomài Freixanet, 1991; Breivik et al., 1994; Wagner and Houlihan, 1994).

Extreme sport has also been viewed as a contradiction to “normal” behaviour, which generally seeks safety and avoids high-risk (Fletcher, 2004). The idea that participants choose to “accept the possibility” of injury or death (Breivik, 1996) contradicts theories such as Maslow (1987) which stress that safety is a primary, innate need. Baudry (1991) writes that extreme sport is paradoxical in nature, as it requires one to contest his/her mortality through a strategy of premeditated suicide. This challenges normative thinking as it infers that extreme sport goes beyond official regulations and safety precautions and can purposefully place the participant in a potentially fatal situation. It implies that extreme sport is dangerous, unregulated and could arguably involve breaking laws or safety regulations, e.g., trespassing is often intrinsically linked to the sport of BASE jumping.

*High Risk* is a key concept in the definition of extreme sport and therefore **Table 1** includes the component of risk of injury and mortality related to a range of sports. High risk is often used interchangeably with extreme sport.

Although terms such as Whiz (Midol, 1993), Post-modern, Post-industrial, New sport, Unconventional, and Non-traditional and Panic sport, have been used in the past (Rinehart and Sydnor, 2003) the most prevalent terms perceived as representing extreme sport which are subsequently outlined in this review, are: Alternative, Action, Adventure, Lifestyle, Media Driven, and Individualism.

## Is “Extreme Sport” Just an “Alternative Sport” to “Traditional Sport?”

In North America, the word “alternative” is popularly used to denote any sport not American (Humphreys, 1997; Rinehart and Sydnor, 2003) whereas researchers such as Kay and Laberge (2002) have used the term “alternative sport” in a more universal way to describe sports which are non-traditional sports. The difficulty in using this term as an all-encompassing word for extreme sport is that many sports are “alternative” as they challenge the societal concept of what is the norm but not all “alternative” sports are extreme (Jarvie, 2006). Arguably then the term “alternative” can be merely a transient term until the “alternative” sport becomes mainstream, thus conventional. For example, Howe (1998) suggests that alternative sport depends on the masses for its continued existence, for once alternative sport becomes commercial and popularised by the public it becomes mainstream. Rinehart and Sydnor (2003) recognise this as an irony as they acknowledge that what is alternative quickly becomes conventional so a dynamic definition of extreme sport, due to perceptual changes, would be needed. Arguably then, in view of this, the term “extreme sport” is therefore considerably more accurate than the widely used term “alternative sport.”

## Is “Extreme Sport” the Same as “Action” or “Adventure” Sport?

“Action” sports are an assortment of “risky, individualistic and alternative sports such as skateboarding, BMX biking, surfing, street luge, wakeboarding, and motor cross” (Bennett and Lachowetz, 2004). Griffith (2002) explores the definition of action sports as something that has evolved from the broader sporting culture of surfing, skating, snowboarding and wakeboarding. Advertising companies employ the term as an effective association in creating a “cool” desirable, brand.

Winged suit jumper Chris “Douggs” McDougall prefers the term “adventure sport” to “extreme sport” because every time he participates he feels that he is going on a cool adventure (O’Neil, 2017). The term adventure sport is used a great deal commercially. The Mintel Report (2003a) noted a division in the reporting of sporting holidays as either hard or soft adventure, whereby “hard” adventure holidays promote risk, danger, challenge and an adrenalin rush. These types of holidays offer caving, mountaineering, white water rafting and skydiving. Adventure sport may be a commonly used term amongst holiday promoters as the words themselves denote excitement and fun. Adventure sports also depict lifestyle sports as they are a leisure time pursuit with not only physical, but also mental exercise. They are journeys through which participants face their own limits of fear, exhaustion and risk, however, they are based more on individual achievement than many traditional sports. For example, the competition element between individuals could be lacking though it is evident that “competition” may exists between the participants and their environment. Adventure sport is a term commonly used in the tourism industry, however, when searching for a universal term for the sake of academic research it is limiting as sports such as BASE jumping or Stunt Cycling or Drag Racing would not readily fit into this category.

The key term, *natural environment* emanating from action and adventure research is another component placed in **Table 1** so that the researchers could see whether there is a pattern of words which emerge to formulate the start of definition and this academic debate. In addition, the idea of competition versus self-evaluation found in the above literature was also included.

## Is “Extreme Sport” Just a Lifestyle Sport?

The term “lifestyle sport” as utilised in the Mintel Report (2003b) identifies specific sports through an examination of the link between the participants, the activity and the environment. Their popularity represents a bottom-up approach steeped in grass root participation that is welcoming to all who want to participate. Those who have been alienated by traditional school-based and institutional sport are often attracted to lifestyle sports (Wheaton, 2004). Affiliation provides participants with membership into an exclusive club – which includes equipment, clothes, like-minded people, books and web sites and can create a social group and sub-culture. In essence, it is sharing the enthusiasm for sport with others who share the same passions and yearn for the same excitement. There are commonalities between “Lifestyle” and “Extreme” sports whereby participants have a sense of camaraderie as they learn from each other via a dress code (e.g., Surfers, Skiers, Skateboarders), specialist web sites as well as the need for specialised equipment.

Tomlinson et al. (2005) considered the “lifestyle” definition to be ambiguous and problematic. They described lifestyle as a way in which individuals interpret their lives for themselves and for others. Using that definition to distinguish between sports would require a differentiation between each person’s motivations for participation in sport. Lifestyle sports relate to those sports pertaining to individual or personal factors. It is more of a descriptor than an encompassing way to describe a variety of sports. Those that do undertake extreme sport, however, may agree that participation in extreme sport does become a lifestyle of sorts when they are with others who are also engaging in their sport.

Alternatively, high risk can refer to spatial dimensions, based on “extreme locations – wilderness, remoteness, the forbidden” (Tomlinson et al., 2005). Sport where participants compete with the natural elements in locations with snow, hills, canyons, islands, mountains, rivers, or volcanoes would fit into the category of “high risk” sport, e.g., extreme skiing and white water rafting. As mentioned previously these are also sometimes referred to as “adventure” sports. Brymer and Oades (2009) labelled “high risk” sport as being undertaken in the natural environment, however, not all “high risk” sports meet this criterion. BMX, Drag Racing and Big Air Snowboarding, for example, take place on a man-made track and Skateboarding can be performed inside or outside and may involve a ramp designed and manufactured specifically for the performance of sport. So although performance in a natural environment is true for some “high risk” sports and could be true for many extreme sports it is not categorically accurate for all extreme sports.

## Is “Extreme Sport” Media Driven Terminology?

So is extreme sport merely a new term for high-risk sport and if so where did the term “extreme sport” emanate from? Arguably what constitutes extreme sport has been predominantly media led (Kay and Laberge, 2002), whereby the term extreme sport has been based on the sale-ability in promoting non-traditional sport to the media and for the increase in consumerism and corporate interest. Sponsorships, endorsements, TV marketing and advertising all utilise the term “extreme sport” for these reasons. For example, the 2014 Winter Olympics became the first games to classify such events as Snowboarding, Ski Jumping, Freestyle Skiing, Skeleton, Luge, Kayaking, and Windsurfing under “extreme sport” umbrella. The 2018 games included as extreme sport events Big Air Snowboarding, Mixed Alpine Skiing, and Mass Start Speed Skating. The 2020 Olympic games in Tokyo has approved the inclusion of the extreme sports of surfing, rock climbing and skateboarding (Herreria, 2016). Adaptive sport is pursuing extreme sport as a cultural norm with the characteristics of increasing heart rate, adrenalin rush, and action sport (Denq and Delasobera, 2018). Interestingly, the term “extreme sport” is probably the most prevalent term used in the media for these types of sports.

## Does “Extreme Sport,” Include a Component of Individualism?

“Extreme sport” can be a way of striving for self-actualisation. Those who are self-actualised according to Maslow (1987) have a sense of self-acceptance and the thrill in living for the moment. Researchers examining these terms for “extreme sport” have focused on the psychological motivation the participants need to find “self-actualisation and spiritualism” (Borden, 2001), promote a “positive personal change” (Brannigan and McDougall, 1983) or fulfil the desire of a “powerful life wish” (Brymer and Oades, 2009).

Robinson (1992, p. 99) viewed “extreme sport” as an activity based on both cognitive and emotional components, as a “a variety of self-initiated activities that generally occur in natural-environment settings and that, due to their always uncertain and potentially harmful nature, provide opportunity for intense cognitive and affective involvement.” Tomlinson et al. (2005) also recognised an “emotional dimension” within “extreme sport” which can be identified as a sensation of wholeness. This is akin to the concept of flow which Csikszentmihalyi (1975) described as a conscious state of being completely absorbed in a situation or sport. The sense of elation and peace experienced in “extreme sport” may be the result of a rush of adrenalin and release of endorphins, which are endogenous mood enhancers.

Puchan (2004) suggests that underlying the growth of “extreme sports” are societal factors such as computer games and various websites designed to promote excitement and/or fear. These cultural changes within particular areas of society encourage individuals to test themselves against great odds without having to leave the parameters of their home. However, in an effort to escape what Puchan (2004) calls boredom and mediocrity, individuals search for outlets where the self can be rediscovered. The concept of “extreme sport” as an answer to boredom fits in with the notion of boredom as a factor in Zuckerman’s (1994) subscale of sensation seeking.

Thrill seeker sports participants are typically 24–34 year old males, single and 80% are without children (Sport England, 2015) therefore one could argue that they have ample spare time and are bored with life? Griffith (2002) sees the market of extreme sport as being youth oriented, as a sport that doesn’t require a group or team and therefore open to anyone who wishes to participate. Moreover if “extreme sports” were predominantly youth oriented, then this term makes an immediate assumption that those who participate are all younger adults which is not the case. Most extreme sport participants are on average aged around 30–31 years: e.g., in Triathlon (off road) the average age is 31 years, Windsurfing 30 years and Sport Climbing 30 years (Outdoor Participation Report, 2013). The latest figure by the Outdoor Foundation Topline Report produced by the Physical Activity Council (2016) shows that 56% of all those that participate in outdoor activities are aged between 15 and 44 years. Clearly, from a developmental perspective, this age group is in a period of transition from adolescence into adulthood, therefore arguably there may be an individualistic nature to extreme sport. Moreover it could be viewed in some instances as a modern rite of passage (Groves, 1987). Perhaps part of the appeal of extreme sport is due to its’ challenging nature at a period (in western culture) when the uncertainty of adulthood is approaching, thus further supporting the argument for a strong self or narcissist focus.

Wheaton (2004) discussed this narcissistic focus as a need for isolation. So while, in many cases, traditional sports promote the ideal of teamwork, extreme sports are focused on individual goals: a more personalised way of challenging oneself without an organised winning or losing concept. Here the emphasis is mostly on self-competition through personal challenges and the idea of just “doing it” (Tomlinson et al., 2005). Arguably, for this reason the term “extreme sport” is often synonymous with “individualistic sport” (Puchan, 2004), whereas traditional sport focuses on the challenge of competition, extreme sport focuses on individual achievement.

## Conclusion and Implications

From a scientific perspective there are difficulties when setting out to examine extreme sport due to a lack of consensus on the tenets of extreme sport. One of the aims of this article was to contribute to the literature on extreme sport and enhance the academic debate prescribing a new workable definition for the sporting literature. However, this objective has been problematic as the definition of extreme sport is ill-defined due mainly to a variety of terms having been used interchangeably with little scientific evidence in support, namely extreme, alternative, high risk, action, and lifestyle sports. This lack of consistency in terminology means that those wishing to study this field are forced to create their own criteria as a starting point, often in a less than scientific approach. As definitions are important to the start of evidenced based research or argument, this article focused on examining the terminology commonly used to represent what is generally perceived as “sporting activities outside of the norm” in order to distinguish between the various terms.

When examining the available research, it also became evident that a variety of interchangeable terms are used by the media, e.g., high-risk sport, adventure sport, alternative sport, lifestyle sport, and action sport as well as extreme sport. These terms have been identified and are in use according to the Mintel Report (2003a) on “Sport Activity in the United Kingdom.” Interestingly, each definition or synonymous term also contains components that give insight into the personality and the motivation of “extreme sport” participants. For example, adventure sport infers challenge along with uncertainty, whilst lifestyle sport implies camaraderie.

Tomlinson et al. (2005) concluded that there were “no universally agreed terms to describe the sports (extreme sports), no agreed categorisations through which to order and understand them and little in the way of governance structures to regulate them” (p. 5). Yet extreme sport, because it has yet to be fully defined, has, to some extent, been a created by the media complete with a “marketing strategy, an ethic, a vocabulary, an attitude, and a style” (Kay and Laberge, 2002).

This article proposes another way in which the term “extreme sport” may be considered so that ambiguity within research is reduced in the future. Specifically we argue that “extreme sport” is a predominantly competitive (comparison or self- evaluative) activity within which the participant is subjected to natural or unusual physical and mental challenges such as speed, height, depth, or natural forces. Moreover, an unsuccessful outcome is more likely to result in the injury or fatality of the participant more often than in a “non-extreme sport.” Therefore, it is suggested that incidents of injury/fatality are the defining factors that separate extreme sports from other sports which would fit into the alternative categories listed, i.e., adventure sport, alternative sport, lifestyle sport and action sport. High-risk sport immediately evokes a sense of danger and extremism, activities similar in nature to extreme sport. In this case, for the purpose of scientific investigation, it is suggested that the term “high risk” is not abandoned but that the use of the current new definition proposed incorporates it within a fuller richer definition of Extreme/High Risk Sport.

Extreme or High Risk sport is one of the fastest growing areas in sporting activity this century, due to its nature it attracts the interest of the media worldwide yet, in the context of sport science, it’s definition needs to be needs to be conceptually clear and linguistically accurate and not influenced by terminology promoted by the media. If our scientific endeavours are to be reliable and valuable then our parameters under investigation need to be consistently, clearly defined. A clear definition of “extreme/high risk sport” as contained in this review, employing a system categorised on the number of injuries/fatalities with a sport is, arguably, a solid basis on which to drive the scientific process for future research forward.

## Limitations and Future Research

A limitation of this research is that we have neither discussed nor differentiated between extreme sport as a “sport” or an “activity” furthermore, between recreational or non-recreational as in CEES. Future research will be undertaken to examine a wide range of sports in order to devise a classification system, which ranges from traditional to extreme/high risk sport according to the current working definition which may be based on injury/fatality per capita for each sport in relation to general risk. Indeed, a recent study by Cohen et al. (2018), has shown significant differences in personality traits between athletes engaged in extreme sport (drag racing) and traditional sport (archery). Personality traits are now playing a significant role in the psychological models of rehabilitation and predicted outcomes (Pain and Kerr, 2004). Future research should capitalise on the distinctions made in the present study in examining the role of personality in sport injury and rehabilitation.

Ongoing research conducted by the current authors includes interviewing and surveying those who participate in extreme sport as well as those who don’t participate, in order to gain insight for future directions, with an immediate aim to ascertain where specific sports may lie on a continuum of sports ranging from traditional to extreme/high risk. **Table 1** is a start on examining the categories of risk, extremes in nature (e.g., height, speed, depth) and (elements of the sport definition – competitive, evaluation) being proposed in the definition for extreme sport. The authors will further expand on any arising variables that have not yet been under consideration and on completion of our follow up work the aim is to develop a formula which enables the aspects of each sport to be analysed according to the current working definition, hence enabling evidence based inclusion on a sporting continuum.

A final recommendation is for subsequent researchers to examine sporting categories in line with the current working definition thus building a corpus of evidence by which the debate around what is extreme/high risk can be scientifically judged. This will enable an advance not only into the field of extreme/high risk sport but in sport science research in general.

## Author Contributions

All authors approved the manuscript for publication and agreed to be accountable for all aspects of the work.

## Conflict of Interest Statement

The authors declare that the research was conducted in the absence of any commercial or financial relationships that could be construed as a potential conflict of interest.
